# Supplementary material for: Benchmarking viromics: an in silico evaluation of metagenome-enabled estimates of viral community composition and diversity
Source: PeerJ. 2017 Sep 21;5:e3817. doi: 10.7717/peerj.3817 (PMC5610896; doi:10.7717/peerj.3817)
Supplement: Supplemental Information 1 — Supplementary Figure 1. Characteristics ofmock viral communities. Mock communities generated had different number of genomes (A) and population distribution (B). This led to a range of alpha diversity, as illustrated with Shannon diversity index (C) and Simpson index (D). Mock communities were also designed to display a beta diversity pattern with 4 groups of samples (E and F, BC: Bray–Curtis). The 14 mock communities were designed to cluster into 4 distinct groups, and are colored across all panels according to these 4 groups of significantly similar communities (F, PerMANOVA p-value <0.001). Supplementary Figure2. Schematic of the methods evaluated in this study. (A) Benchmarking of the assemblers, read pre-processing methods, and thresholds on genome coverage and read mapping identity used to calculate abundance matrices. (B) Estimation of the impact of strain heterogeneity on the assembly efficiency. Reference genomes were replaced by populations composed of a set of related strains controlled by 3 parameters. (C) Evaluation of the different normalization methods across the three types of datasets, with varying differences in sequencing depth across samples. For all panels, the different methods tested are indicated for each step, and the method and/or threshold chosen or optimal are highlighted in blue (other tests are colored in gray). The metrics used to identify the optimal methods/thresholds are indicated on the left, in green for metrics to maximize, red for metrics to minimize, and black for metrics to compare to “true” data based on whole communities. QC: quality-controlled. Supplementary Figure3. Influence of assembly software and read curation on genome recovery –dotplots (underlying data for boxplots presented in Figure 1). In these plots, each dot represents the assembly of a single genome in a single sample. (A) Genome recovery (i.e. genome coverage by all contigs, y-axis) by genome coverage (x-axis) for different assemblers (colors). (B) Genome rec [file peerj-05-3817-s001.pdf]

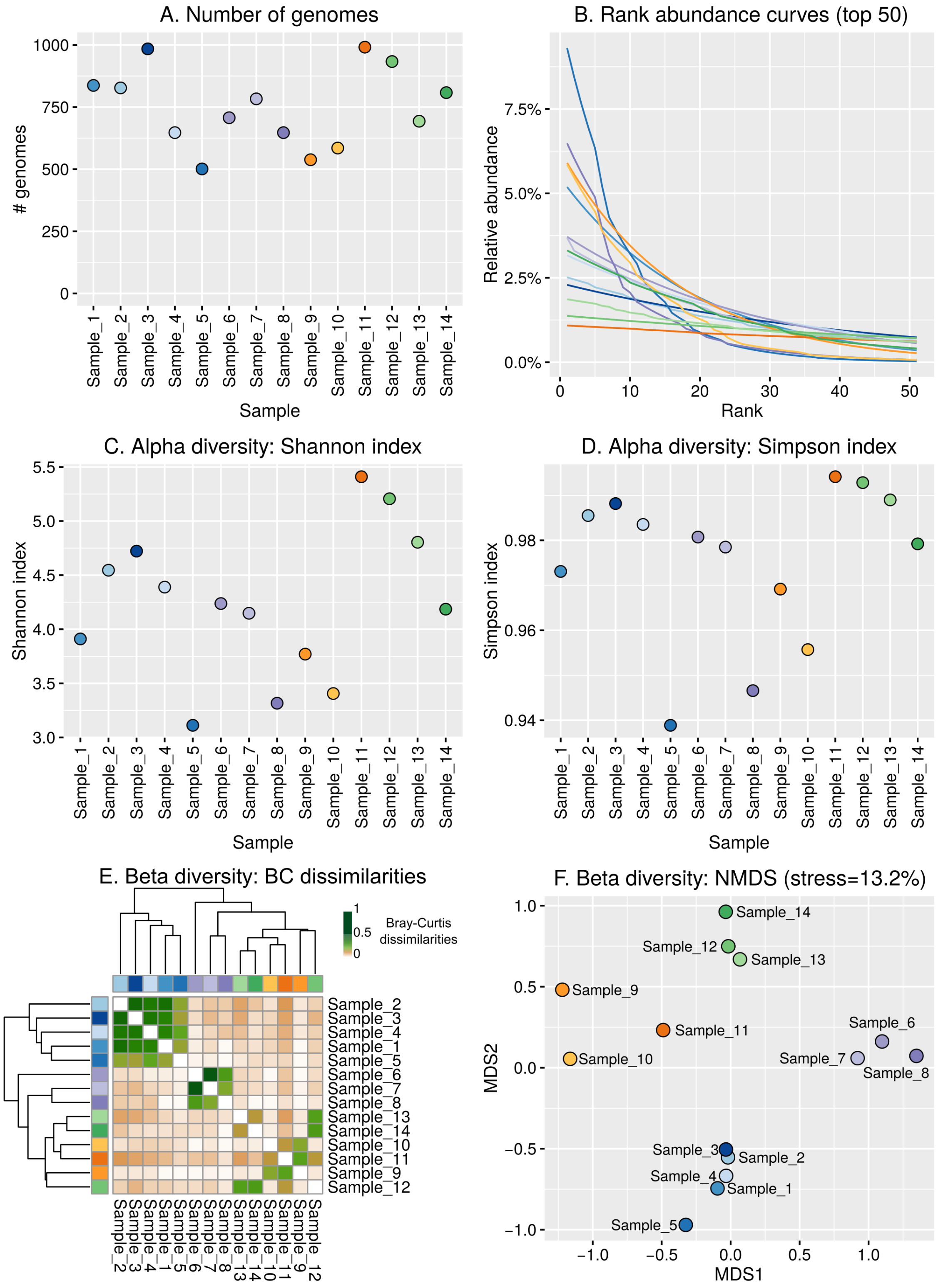

Supplementary Figure 1. Characteristics of mock viral communities. Mock communities generated had different number of genomes (panel A) and population distribution (panel B). This led to a range of alpha diversity, as illustrated with Shannon diversity index (C) and Simpson index (D). Mock communities were also designed to display a beta diversity pattern with 4 groups of samples (E and F, BC: Bray-Curtis). The 14 mock communities were designed to cluster into 4 distinct groups, and are colored across all panels according to these 4 groups of significantly similar communities (panel F, PerMANOVA p-value < 0.001).

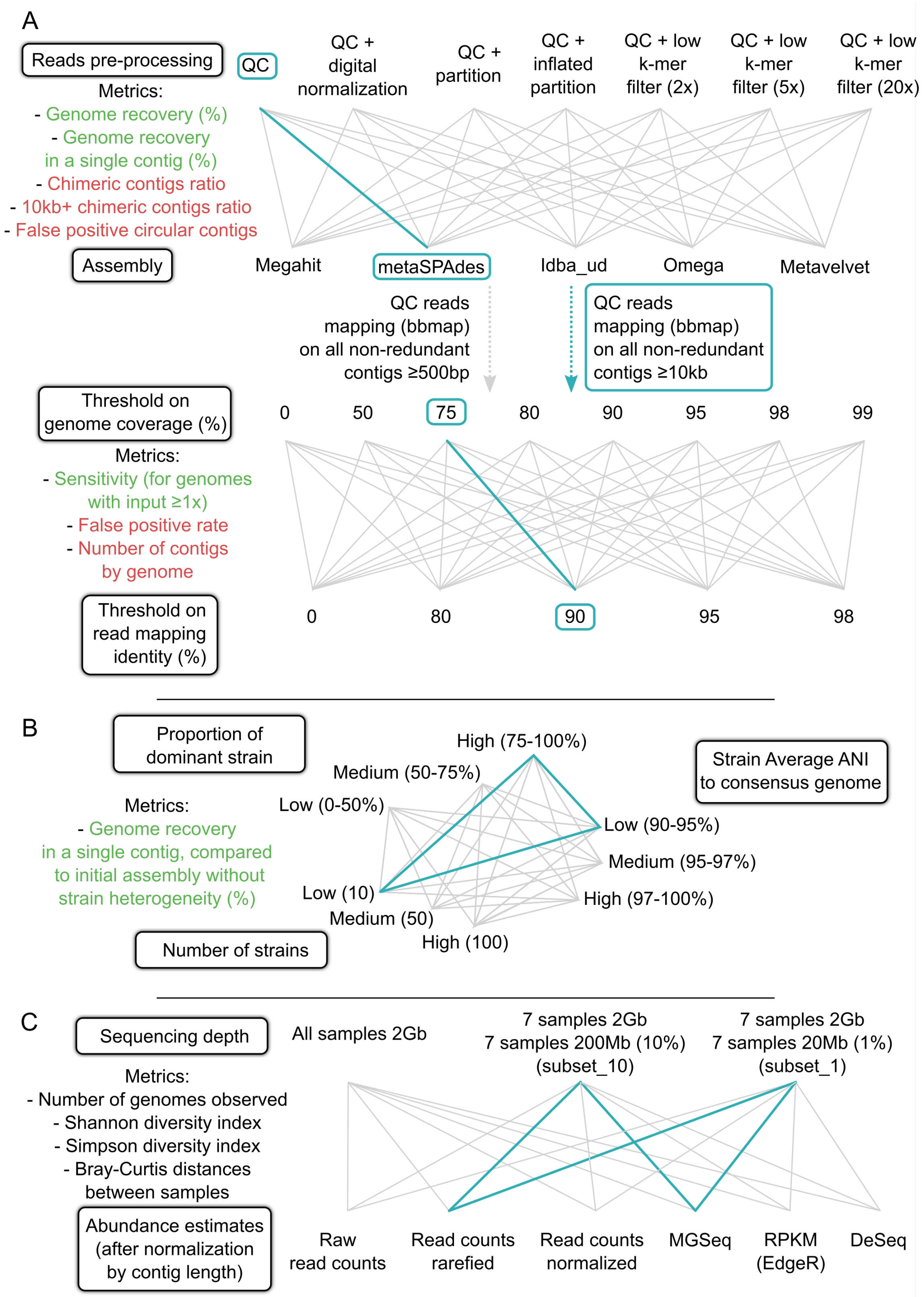

Supplementary Figure 2. Schematic of the methods evaluated in this study. A. Benchmarking of the assemblers, read pre-processing methods, and thresholds on genome coverage and read mapping identity used to calculate abundance matrices. B. Estimation of the impact of strain heterogeneity on the assembly efficiency. Reference genomes were replaced by populations composed of a set of related strains controlled by 3 parameters. C. Evaluation of the different normalization methods across the three types of datasets, with varying differences in sequencing depth across samples. For all panels, the

different methods tested are indicated for each step, and the method and/or threshold chosen or optimal are highlighted in blue (other tests are colored in gray). The metrics used to identify the optimal methods/thresholds are indicated on the left, in green for metrics to maximize, red for metrics to minimize, and black for metrics to compare to “true” data based on whole communities. QC: quality-controlled.

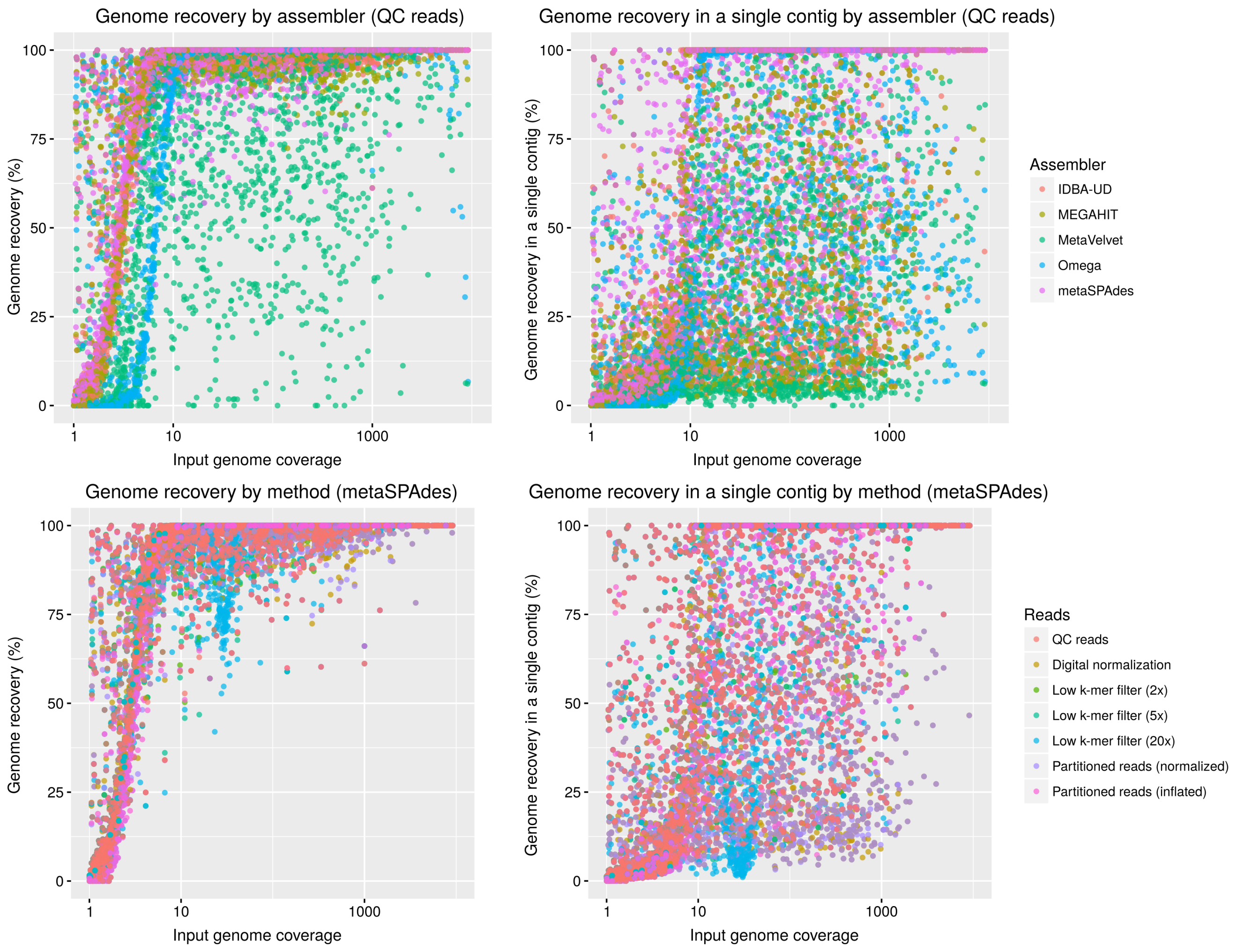

Supplementary Figure 3. Influence of assembly software and read curation on genome recovery – dotplots (underlying data for boxplots presented in Figure 1). In these plots, each dot represents the assembly of a single genome in a single sample. A. Genome recovery (i.e. genome coverage by all contigs, y-axis) by genome coverage (x-axis) for different assemblers (colors). B. Genome recovery in a single contig (i.e. genome coverage by the largest assembled contig, y-axis) by genome coverage (x-axis) for different assemblers (colors). C. Genome recovery (i.e. genome coverage by all contigs, y-axis) by genome coverage (x-axis) for different read curation methods (colors). B. Genome recovery in a single contig (i.e. genome coverage by the largest assembled contig, y-axis) by genome coverage (x-axis) for different read curation methods (colors).

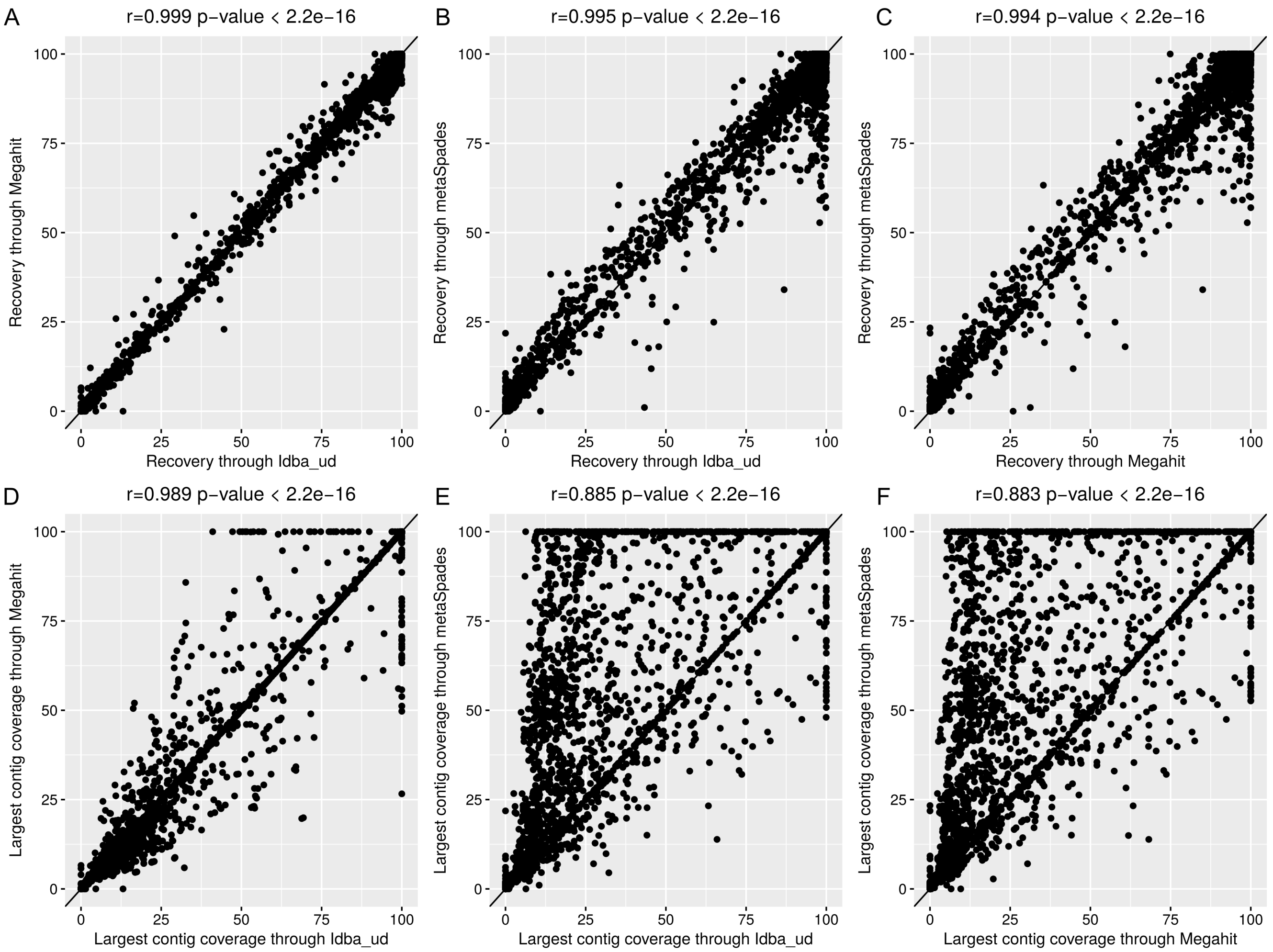

Supplementary Figure 4. Correlation between assembly results of different assemblers. Top panels display the correlations of genome recovery (i.e. genome coverage by all contigs) for each genome between Megahit and Idba_ud (A), metaSPAdes and Idba_ud (B) and metaSPAdes and Megahit (C). Bottom panels display the correlations of genome recovery in a single contig (i.e. genome coverage by the largest assembled contig) for each genome between Megahit and Idba_ud (D), metaSPAdes and Idba_ud (E) and metaSPAdes and Megahit (F).

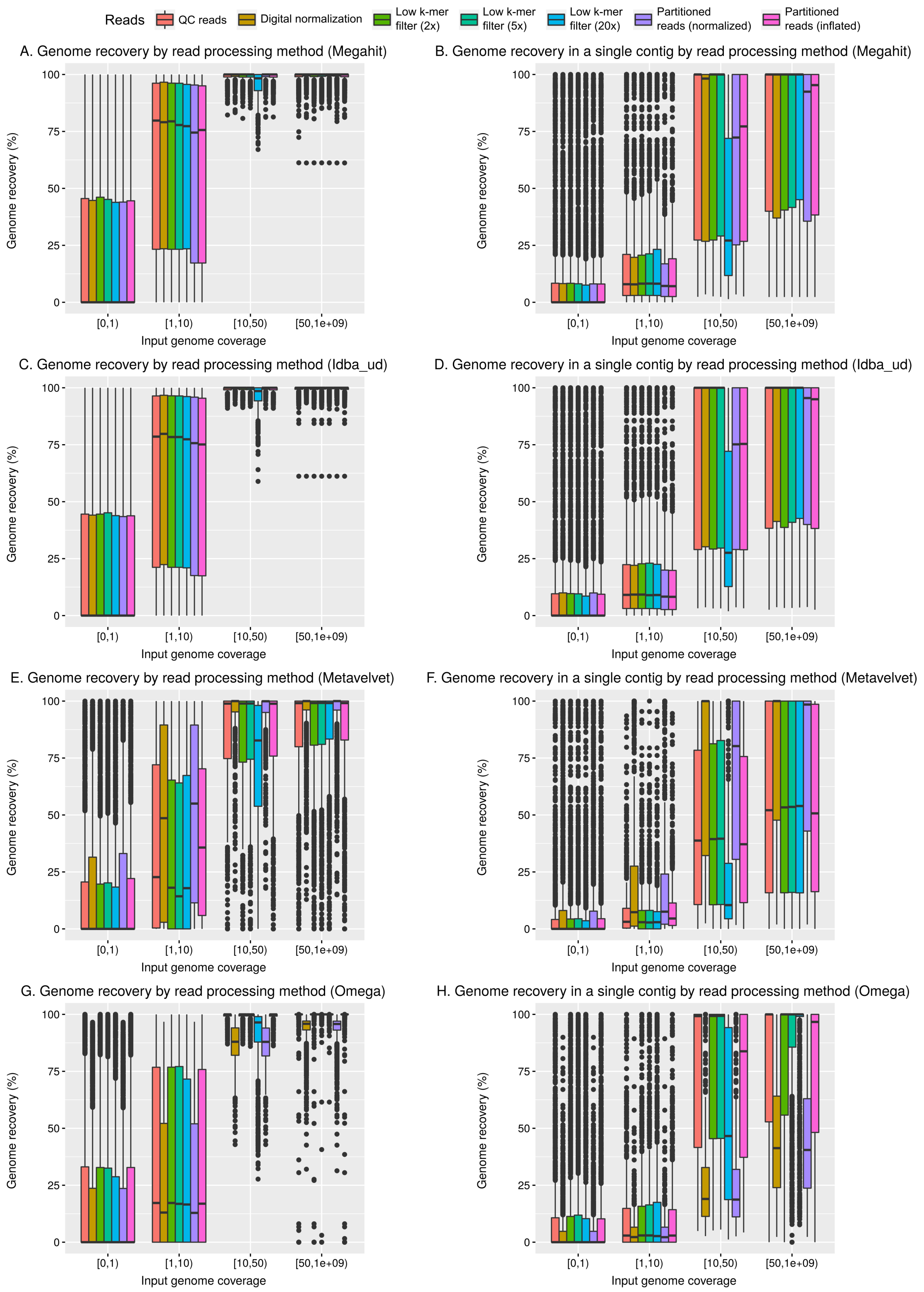

Supplementary Figure 5 Influence of read curation on genome recovery for different assemblers. The assemblers used here are Megahit (A, B), Idba_ud (C, D), Metavelvet (E, F), and Omega (G, H). For each assembler, the genome recovery (i.e. genome coverage by all contigs, y-axis) by genome coverage (x-axis) for different read curation methods (panels A, C, E, and G) as well as genome recovery in a single contig (i.e. genome coverage by the largest assembled contig, y-axis) by genome coverage (x-axis) for different read curation methods (panels B, D, F, and H) are displayed. Similar data are displayed for metaSPAdes in Fig. 1 panels C and D.

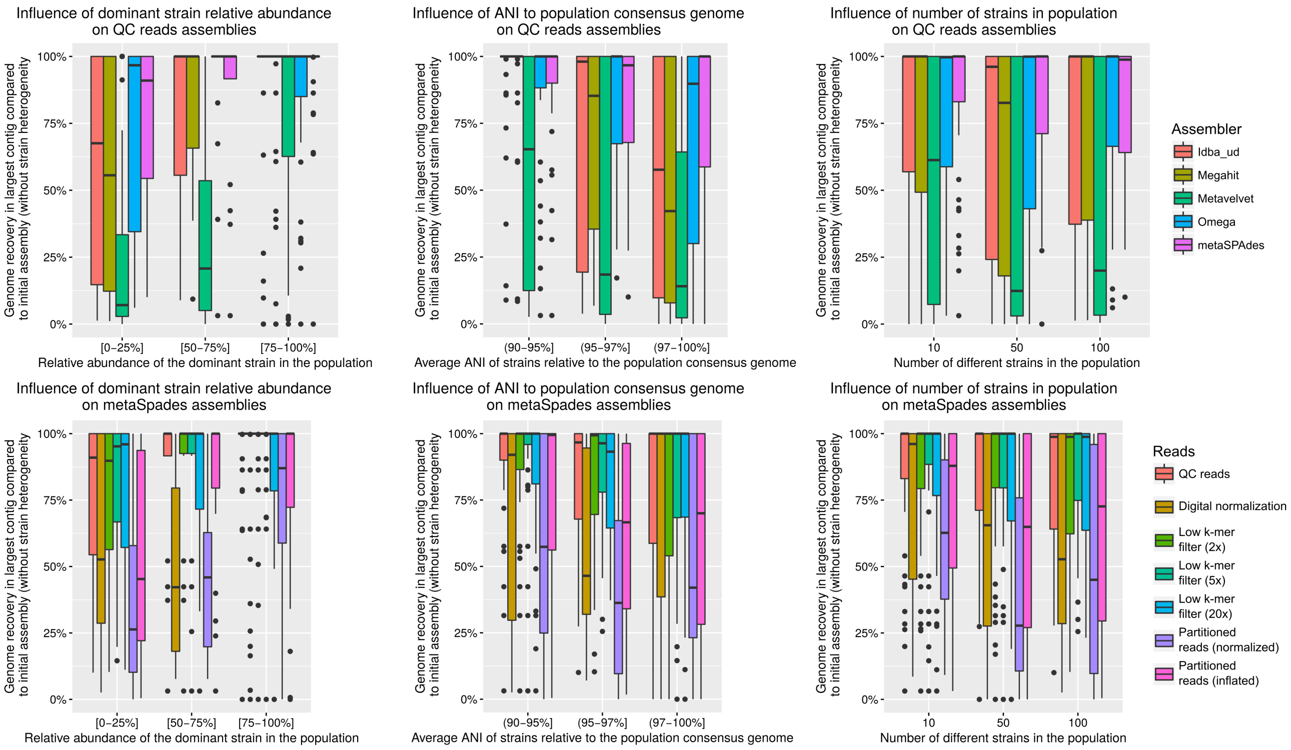

Supplementary Figure 6. Influence of strain-level diversity on assembly efficiency. These tests were computed on one mock community (Sample_1), where each reference genome was replaced with a set of related strains with varying divergence and relative abundances. In each plot, the y-axis represents the ratio between the largest contig assembled for a genome when strain heterogeneity is introduced and the same parameter without strain heterogeneity (i.e. previous assemblies of the same Sample_1). Plots on the top row display the differences in QC reads assemblies between assemblers, while plots on the bottom row show differences between different reads processing for metaSPAdes assemblies. Populations are grouped based on the different parameters controlling strain heterogeneity, i.e. relative abundance of the dominant strain (left), divergence of the strains (middle), and number of strains in the population (right).

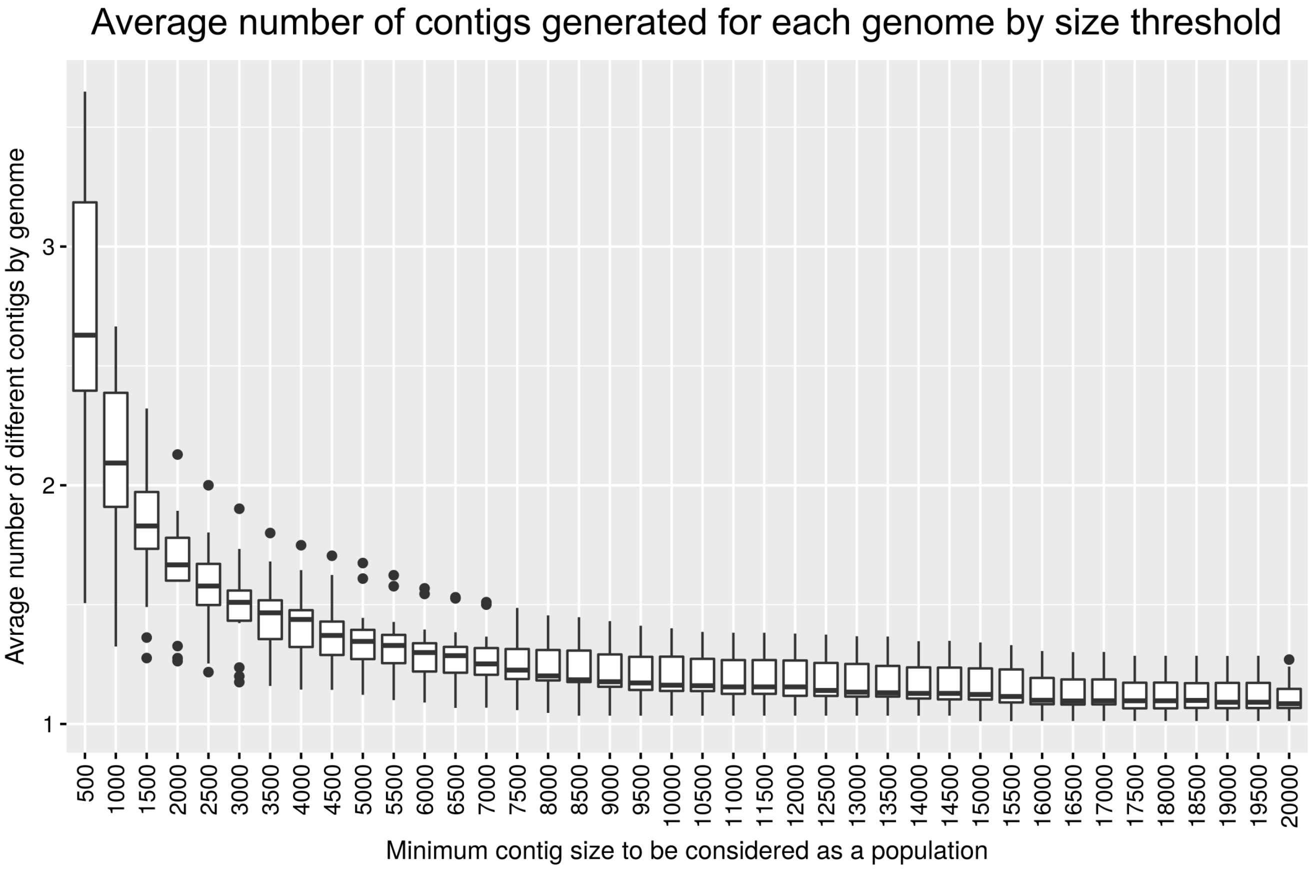

Supplementary Figure 7. Number of population contigs detected for each input genome depending on the minimum contig size threshold for inclusion in population pool. The threshold on contig size used for inclusion in population contigs pools (in bp) is displayed on the x-axis. The distribution of average number of contigs for a given genome (across the samples where this genome was covered ≥ 1x) is displayed on the y-axis.

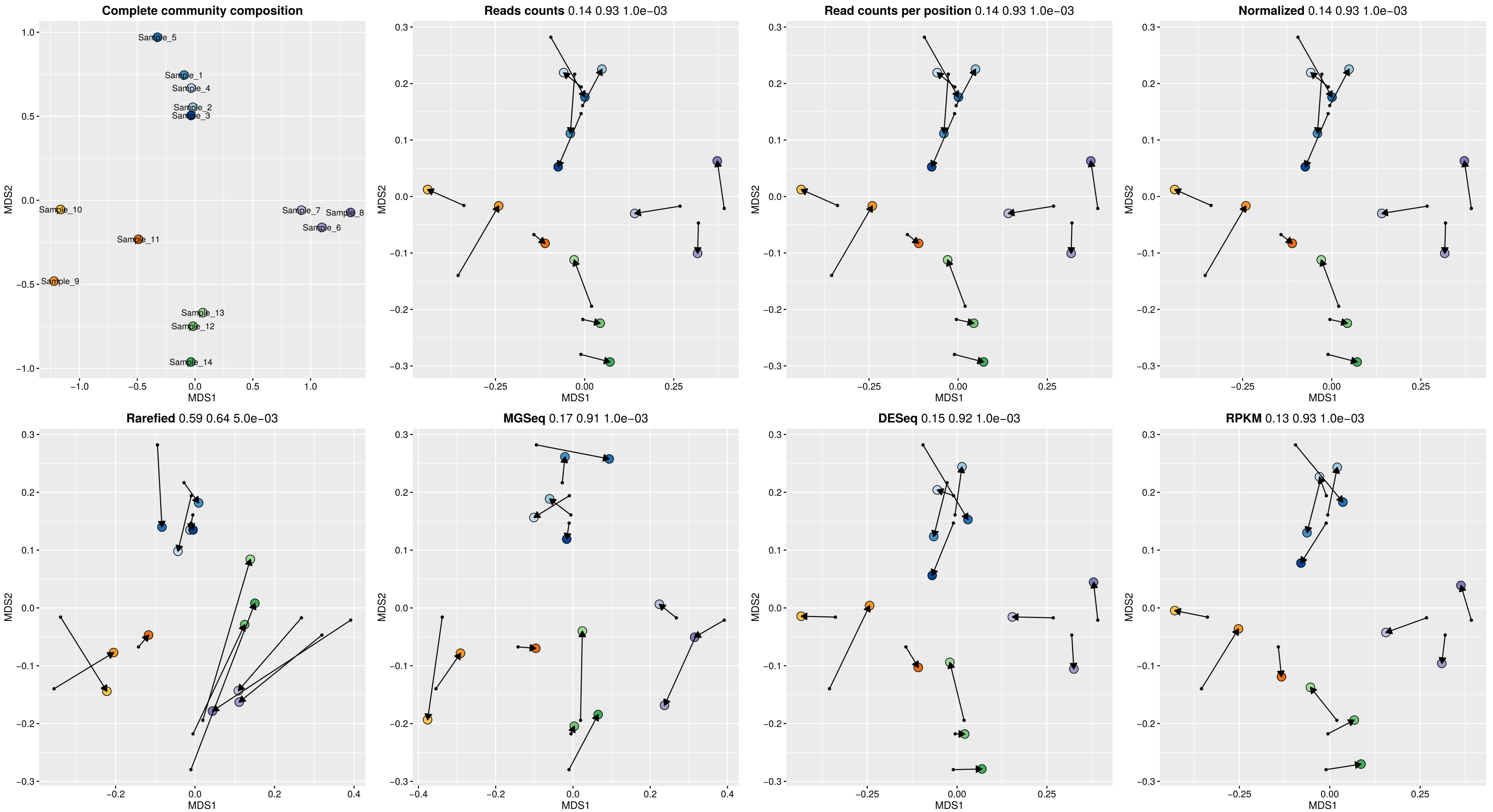

Supplementary Figure 8. Comparison of NMDS based on viral population counts and the reference mock community composition (reference in top left panel). The different NMDS were computed from the viral population count matrices normalized with the different methods tested in the manuscript (in bold), from the dataset including 6 samples strongly under-sequenced (subset_1, i.e. half of the datasets subsampled at 1%). For each NMDS, the sum of square difference, scaling factor, and significance of the correlation to the reference NMDS is indicated in the plot title (calculated with the function protest from the R package vegan). Samples are colored as in Fig. S1, and an arrow is used to illustrate the difference between the original sample placement in the reference NMDS and the new placement in the NMDS derived from population contigs.
